# Tools for Evaluating the Content, Efficacy, and Usability of Mobile Health Apps According to the Consensus-Based Standards for the Selection of Health Measurement Instruments: Systematic Review

**DOI:** 10.2196/15433

**Published:** 2021-12-01

**Authors:** Antonio Muro-Culebras, Adrian Escriche-Escuder, Jaime Martin-Martin, Cristina Roldán-Jiménez, Irene De-Torres, Maria Ruiz-Muñoz, Manuel Gonzalez-Sanchez, Fermin Mayoral-Cleries, Attila Biró, Wen Tang, Borjanka Nikolova, Alfredo Salvatore, Antonio Ignacio Cuesta-Vargas

**Affiliations:** 1 Grupo Clinimetría (F-14), University of Málaga Málaga Spain; 2 Instituto de Investigación Biomédica de Málaga (IBIMA) Malaga Spain; 3 Physical Medicine and Rehabilitation Unit Regional University Hospital of Malaga Málaga Spain; 4 Mental Health Unit Regional University Hospital of Malaga Málaga Spain; 5 ITWare Budapest Hungary; 6 Faculty of Science and Technology, Bournemouth University Bournemouth United Kingdom; 7 Arthaus Skopje Republic of North Macedonia; 8 SensorID Snc Boiano Italy; 9 School of Clinical Science, Faculty of Health Science, Queensland University Technology Queensland Australia

**Keywords:** mobile health, mHealth, eHealth, mobile apps, assessment, rating, smartphone, questionnaire design, mobile phone

## Abstract

**Background:**

There are several mobile health (mHealth) apps in mobile app stores. These apps enter the business-to-customer market with limited controls. Both, apps that users use autonomously and those designed to be recommended by practitioners require an end-user validation to minimize the risk of using apps that are ineffective or harmful. Prior studies have reviewed the most relevant aspects in a tool designed for assessing mHealth app quality, and different options have been developed for this purpose. However, the psychometric properties of the mHealth quality measurement tools, that is, the validity and reliability of the tools for their purpose, also need to be studied. The Consensus-based Standards for the Selection of Health Measurement Instruments (COSMIN) initiative has developed tools for selecting the most suitable measurement instrument for health outcomes, and one of the main fields of study was their psychometric properties.

**Objective:**

This study aims to address and psychometrically analyze, following the COSMIN guideline, the quality of the tools that are used to measure the quality of mHealth apps.

**Methods:**

From February 1, 2019, to December 31, 2019, 2 reviewers searched PubMed and Embase databases, identifying mHealth app quality measurement tools and all the validation studies associated with each of them. For inclusion, the studies had to be meant to validate a tool designed to assess mHealth apps. Studies that used these tools for the assessment of mHealth apps but did not include any psychometric validation were excluded. The measurement tools were analyzed according to the 10 psychometric properties described in the COSMIN guideline. The dimensions and items analyzed in each tool were also analyzed.

**Results:**

The initial search showed 3372 articles. Only 10 finally met the inclusion criteria and were chosen for analysis in this review, analyzing 8 measurement tools. Of these tools, 4 validated ≥5 psychometric properties defined in the COSMIN guideline. Although some of the tools only measure the usability dimension, other tools provide information such as engagement, esthetics, or functionality. Furthermore, 2 measurement tools, Mobile App Rating Scale and mHealth Apps Usability Questionnaire, have a user version, as well as a professional version.

**Conclusions:**

The Health Information Technology Usability Evaluation Scale and the *Measurement Scales for Perceived Usefulness and Perceived Ease of Use* were the most validated tools, but they were very focused on usability. The Mobile App Rating Scale showed a moderate number of validated psychometric properties, measures a significant number of quality dimensions, and has been validated in a large number of mHealth apps, and its use is widespread. It is suggested that the continuation of the validation of this tool in other psychometric properties could provide an appropriate option for evaluating the quality of mHealth apps.

## Introduction

### Background

Nowadays, in the age of digital content, people, regardless of age group, have access to mobile and smart devices (eg, phones, tablets, and smart televisions), or special devices with the possibility of internet connection. In dedicated (iOS and Android) app stores (Apple App Store and Google Play Store), there are thousands of apps with a vast number of functions, and this number is increasing every day. According to a new report by Grand View Research, Inc [1], the mobile health (mHealth) app market size is expected to reach US $149.3 billion by 2028 and is expected to register a compound annual growth rate of 17.7% over the forecast period. In these app catalogs, mHealth apps are a very important field, and there has been a growing interest from users in the last few decades. Some studies report that up to 34% of mobile phone owners have at least one health app installed on their device [2]. The World Health Organization [3] described the term mHealth as *the use of mobile wireless technologies for health,* being a subset of eHealth, which is described as *the use of information and communications technology in support of health and health-related fields*. The World Health Organization [3] also highlighted the relevance of digital health interventions to address health needs, remarking that they should always be used as an aid and an improvement for health systems, not as a substitute.

The variety of features in apps available on different platforms or cross-platforms is wide. Therefore, many of the mHealth apps are (1) simply a catalog of recommendations; some of them work as a (2) follow-up tool, complementing an intervention program, whereas other mHealth apps are (3) connected to dedicated sensors to offer information about health signals or health status.

Most of these mHealth apps enter the market with limited filters or controls that usually do not consider aspects such as the veracity of its content and their effectiveness as relevant [4]. According to a previous study, only a small percentage of available apps in some health fields have referred to medical professional involvement in their development or content [5]. Both, apps that users use autonomously and those that can be directly recommended by clinicians to their patients require a prior study that investigates their evidence to minimize the risk of using the apps that do not work or that may even cause harm [6,7]. The development of these studies and analyses have been described in academic contexts, but its execution is not always easy in commercial apps [5]. Some experts attribute this fact to a much slower pace of academic research than that of app development, which can result in long delays in the diffusion of apps in commercial markets and among users [8]. Thus, many of the apps are only rated by the general subjective perception of users with vague rating tools, such as numerical or star-based scores from 1 to 5.

Many attempts have been made to develop effective and practical validation tools to measure the quality of mHealth apps. The quality-based concept has been interpreted in different ways according to each author and field, evaluating or resulting in different components [9]. Some of the first attempts used existing generic tools, such as the System Usability Scale (SUS) [10], to measure the usability, that is, the ease of use, of systems. This tool was developed in 1986 to allow a quick and basic measurement of the usability of any system and is still used in many studies despite being 30 years old. The increase in the use of new technologies, such as smartphones and health apps, brought the need to develop new types of mHealth apps with specific measurement tools that adapt the existing ones.

Previous studies have reviewed which methods have been used to assess the quality of mHealth apps [11,12], determining which aspects could be the most relevant in a tool designed for this purpose [2]. In general, quality evaluation methodologies can be divided into 2 categories: (1) methodologies based on the downloaded app content (using a predefined list of requirements that the app should contain, assessing the inclusion of evidence-based content, and assessing the usability of predefined app functions) and (2) methodologies that are content-independent and the app does not need to be downloaded (using app market or website assessment tools, users’ reviews and ratings, and other assessment methods such as the analysis of the app description or a medical professional involvement in the app) [11]. Similarly, a previous review suggested that the essential contents to be evaluated can be grouped into 4 categories: (1) content analysis (coding and qualitatively evaluating the app content); (2) usability testing (evaluating whether the app works correctly and its ease of use); (3) observational studies (that can be used to assess app use and satisfaction and to predict its usefulness in certain contexts); and (4) efficacy testing (assessing whether the app achieves meaningful effects in previously determined outcomes) [2]. Other areas of interest in this quality evaluation may include exploring the technical functions of the app, the management of security and privacy of user data, and how the developer will use these or developer transparency [2]. Because the measurement tools do not measure all the dimensions and properties of an mHealth app, a prior analysis is necessary to select the appropriate tool for each purpose.

In addition to the inclusion of the previously proposed aspects, it is important to determine the validity of these instruments, that is, the ability to properly assess what they intend to assess. Therefore, it is relevant for any measurement tool to study its psychometric properties. Psychometric properties are different concepts related to the validity and reliability of the instruments, each helping us to determine whether a tool adequately does what it was designed to do according to essential aspects. The development of psychometrics has provided the possibility of knowing the existence of individual differences in the use of measurement tools and their quantification [13,14]. In 2005, the Consensus-based Standards for the Selection of Health Measurement Instruments (COSMIN) started to develop practical tools for selecting the most suitable measurement instrument in research and clinical practice to improve the selection of outcome measurement instruments for health outcomes. One of the main fields of study was the psychometric properties of the assessment tools. Psychometric analysis, using a wide variety of terminology, has been a source of controversy and confusion for decades. The progress of the COSMIN initiative has improved this aspect through the development of the COSMIN Taxonomy of Measurement Properties. This tool aims to standardize the psychometric criteria necessary to validate patient-reported outcome measures [15,16]. These guidelines were not specifically designed for digital health. However, it is necessary to bring the tools of psychometrics closer to this field to evaluate the measurement instruments used to choose mHealth apps in a clinical context. Therefore, it is essential to integrate this analysis in the field of digital health in their original format and possibly later in a more specific version adapted for this field. In this context, a wide analysis of mHealth assessment tools, following this guide, seems to be an appropriate method for assessing their quality and suitability, bringing scientific consensus closer to this field of health.

### Objective

The purpose of this systematic review is to address and psychometrically analyze, following the COSMIN guideline, the quality of the tools that are currently used to measure the quality of mHealth apps.

## Methods

This systematic review was conducted in accordance with the PRISMA (Preferred Reporting Items for Systematic Reviews and Meta-Analyses) guidelines [17].

### Data Sources and Searches

PubMed and Embase databases were processed by 2 reviewers (AE-E and AM-C) from February 2019 to December 31, 2019. The following search term combinations were used in PubMed: (*mHealth* OR *mobile* app* OR *health* *technology*) AND (*scale* OR *checklist* OR *score*) AND *app*. The search was extended to all fields. Embase was searched using the following search string: (*health*/*exp* OR *health*) AND *app* AND (*quality*/*exp* OR *quality*). Complementary searches were performed on the reference lists of the reviews and included articles. The outcome selection process was as follows: when a tool was identified, the instrument was specifically searched in the database search engines, to find all the validation studies associated with this tool.

For inclusion, the studies had to be meant to validate a tool (scale, score, index, or questionnaire) designed to evaluate the quality of systems and used to assess mHealth apps.

Studies that included the application of these tools for the evaluation of mHealth apps but that did not include any type of psychometric validation were excluded. In addition, studies that contained self-written questionnaires where authors focused on measuring usability or content of apps without any validation of their use were also excluded because of the lack of reliability.

### Study Selection

The search results were screened by title and abstract by 2 independent authors (AE-E and AM-C). Whenever the information contained in the title and abstract was insufficient, the full text was examined to decide. Full texts of all potentially eligible studies were independently screened by the same reviewers to identify those that met the abovementioned selection criteria. Disagreements were agreed upon by a third reviewer (AIC-V). Finally, the measurement tools included in the selected studies were identified and retrieved.

### Data Extraction

Data from the selected studies were extracted by the same independent reviewers using an extraction form. The extracted information included the original language, cross-cultural adaptations available, number of dimensions, number of items, and the fulfillment of the 10 psychometric characteristics described in COSMIN (internal consistency, reliability, measurement error, content validity, structural validity, hypotheses testing, cross-cultural validity, criterion validity, responsiveness, and interpretability). Any discrepancies identified were discussed and resolved by bringing in a third reviewer (AIC-V) whenever a consensus could not be reached.

### Quality Assessment: Psychometric Characteristics (COSMIN Analysis)

The psychometric characteristics of each of the retrieved tools were analyzed following the COSMIN guidelines [15] based on the COSMIN Taxonomy of Measurement Properties to assess their methodological quality. The COSMIN guideline also offers updated values to consider psychometric properties as sufficient [15]. The definitions provided by COSMIN for all psychometric characteristics are presented in Table 1. We examined 10 psychometric characteristics. First, the content validity of each tool was assessed. According to COSMIN, content validity is considered to be the most important measurement property because it evaluates whether an outcome measurement instrument is relevant, comprehensive, and comprehensible with respect to the construct of interest and target population [15]. Second, the internal structure of the outcome measures was evaluated using structural validity, internal consistency, and cross-cultural validity. Third, the remaining measurement properties were evaluated (reliability, measurement error, criterion validity, hypotheses testing for construct validity, and responsiveness). Finally, the interpretability and feasibility of each measurement tool were evaluated.

**Table 1:** Consensus-based Standards for the Selection of Health Measurement Instruments definitions of domains, measurement properties, and aspects of measurement properties [18].

Domain	Measurement property	Aspect of a measurement property	Definition	
Reliability	N/A^a^	N/A	The degree to which the measurement is free from measurement error	
**Reliability (extended definition)**				The extent to which scores for patients who have not changed are the same for repeated measurement under several conditions: for example, using different sets of items from the same HR-PROs^b^ (internal consistency) over time (test-retest), by different persons on the same occasion (interrater), or by the same persons (ie, raters or responders) on different occasions (intrarater)
	Internal consistency	N/A	The degree of the interrelatedness among the items	
	Reliability	N/A	The proportion of the total variance in the measurements which is due to *true*^c^ differences between patients	
	Measurement error	N/A	The systematic and random error of a patient’s score that is not attributed to true changes in the construct to be measured	
**Validity**				The degree to which an HR-PRO instrument measures the construct it purports to measure
	**Content validity**		The degree to which the content of an HR-PRO instrument is an adequate reflection of the construct to be measured	
		Face validity	The degree to which (the items of) an HR-PRO instrument indeed looks as though they are an adequate reflection of the construct to be measured	
	**Construct validity**		The degree to which the scores of an HR-PRO instrument are consistent with the hypotheses (for instance, with regard to internal relationships, relationships to the scores of other instruments, or differences between relevant groups) based on the assumption that the HR-PRO instrument validly measures the construct to be measured	
		Structural validity	The degree to which the scores of an HR-PRO instrument are an adequate reflection of the dimensionality of the construct to be measured	
		Hypotheses testing	Idem construct validity	
		Cross-cultural validity	The degree to which the performance of the items on a translated or culturally adapted HR-PRO instrument are an adequate reflection of the performance of the items of the original version of the HR-PRO instrument	
	Criterion validity	N/A	The degree to which the scores of an HR-PRO instrument are an adequate reflection of a *gold standard*	
**Responsiveness**				The ability of an HR-PRO instrument to detect change over time in the construct to be measured
	Responsiveness	N/A	Idem responsiveness	
Interpretability^d^	N/A	N/A	Interpretability is the degree to which one can assign qualitative meaning, that is, clinical or commonly understood connotations, to an instrument’s quantitative scores or change in scores	

^a^N/A: not applicable.

^b^HR-PRO: health-related patient-reported outcome.

^c^The word *true* must be seen in the context of the classical test theory, which states that any observation is composed of 2 components: a true score and an error associated with the observation. *True* is the average score that would be obtained if the scale were given an infinite number of times. It refers only to the consistency of the score and not to its accuracy.

^d^Interpretability is not considered a measurement property but is an important characteristic of a measurement instrument.

The information obtained from the validation of the psychometric criteria was used in 2 ways. First, the number of properties described in the COSMIN guideline that were validated was quantified. Second, the meaning of the values obtained for each property was analyzed.

### Measured Dimensions

The number and content of the measured dimensions were identified to facilitate the characterization of each measurement tool. In addition, the number of items was retrieved to determine tool length.

## Results

### Selection of Studies

**Figure 1:**
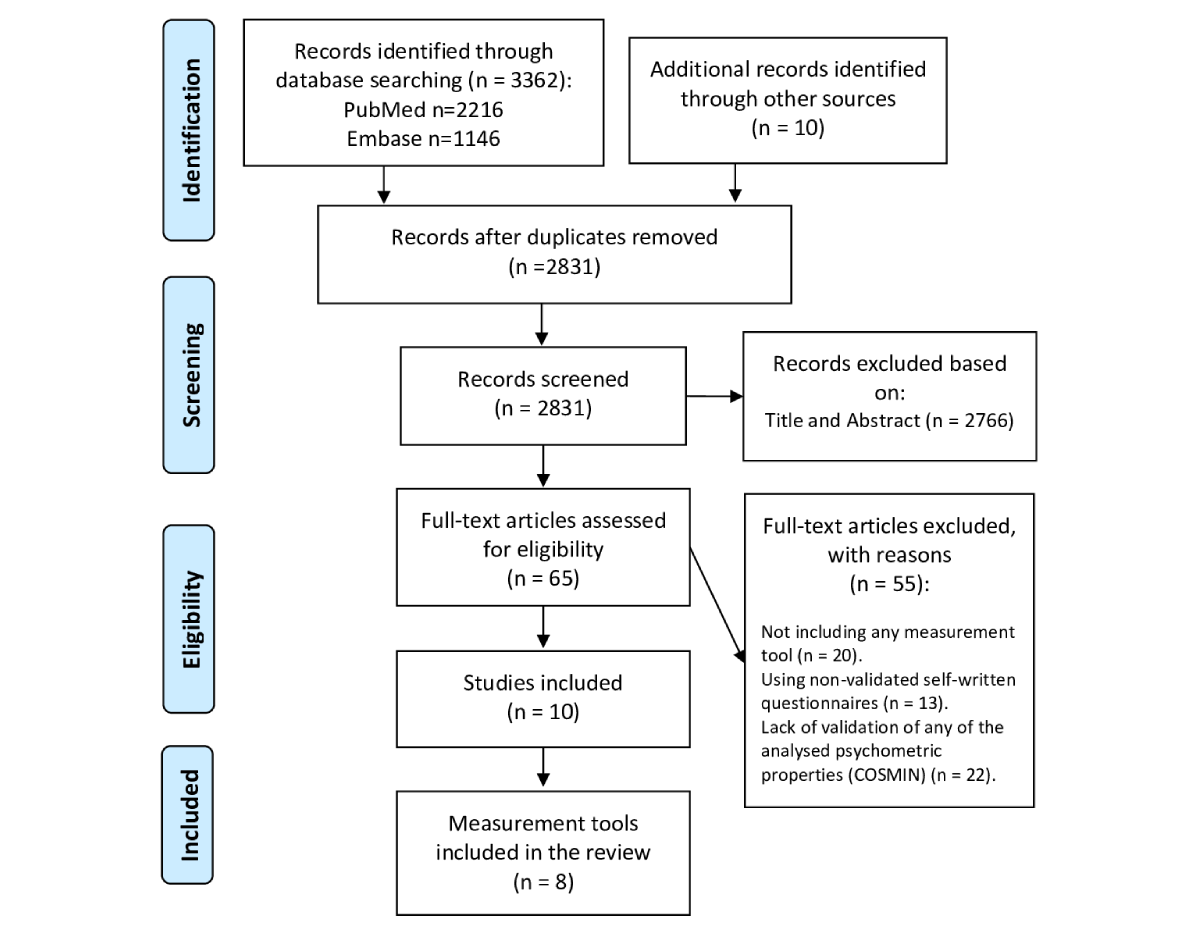
Flowchart of the selection process. COSMIN: Consensus-based Standards for the Selection of Health Measurement Instruments.

The literature research identified 3372 articles, of which 2831 remained after deleting duplicates. From these, 65 studies were selected as potentially eligible after reading the title and abstract (full texts of the studies were retrieved in the case of doubt). We excluded 20 studies because they did not include any measurement tool, and 13 studies were excluded because of the use of nonvalidated self-written questionnaires. After analyzing the psychometric characteristics, 22 studies were excluded because of the lack of validation of any of the psychometric properties recommended by the COSMIN guideline. Therefore, only 10 studies were finally included in the review (Figure 1), including the development process or analysis of 8 measurement tools, with some of these different versions of the same original tool. Therefore, 2 tools (Mobile App Rating Scale [MARS] and mHealth Apps Usability Questionnaire [MAUQ]) have different versions for users and professionals. In addition, the MAUQ provides different versions for interactive or stand-alone mHealth apps.

### Measurement Tools

The oldest scale identified was the SUS, which was developed in 1986, whereas the newest was the MAUQ, which was developed in 2016. In total, 8 tools were identified, some of which were different versions of the same original tool.

#### Usability

As described in the Introduction section, the SUS tool was initially developed to evaluate the usability of engineering and electronic office systems. Nowadays, it is used to evaluate many products and services, such as software, webpages, or mobile apps. It focuses on measuring usability using a Likert scale of 10 elements [10]. It has been adapted to multiple languages, such as Portuguese, Spanish, French, German, Persian, and Malay, and it is considered a highly reliable tool [19-22].

Similarly, 3 other tools included were specifically designed to assess usability [23-25]. First, the tool *Measurement Scales for Perceived Usefulness and Perceived Ease of Use* was developed in 1989 to measure the usability of computer systems [23]. As its name suggests, this tool incorporates 2 scales to evaluate the perception of 2 aspects of usability: usefulness and ease of use. Second, the Health Information Technology Usability Evaluation Scale (Health-ITUES) questionnaire was one of the first questionnaires to focus specifically on health areas [24]. Initial attempts to develop and value Health-ITUES were conducted using a web-based communication system that supported nurse staffing and scheduling [26]. However, its use in an mHealth app was not validated until a few years ago [27]. Finally, the MAUQ questionnaire exclusively focuses on the usability aspects of apps [25]. This questionnaire provides 4 versions, depending on whether it is used by a health professional or by a patient and whether it is intended to analyze an interactive or stand-alone app (interactive app for patients, interactive app for health care providers, stand-alone app for patients, and stand-alone app for health care providers). However, only the patient’s versions have been validated and are therefore included in the analysis of this review, as shown in Table 2.

**Table 2:** Main characteristics of the mobile health apps quality measurement tools included in this review.

Measurement tool	Validation	Year	Language	Cross-cultural adaptation available	Dimensions of the measurement tool	Number of items
Mobile App Rating Scale	60 mental health apps	2015	English	Italian and Spanish	5: engagement; functionality; esthetics; information quality; and subjective app quality	23
iSYScore index	257 health apps	2016	Spanish	—^a^	3: popularity and interest; trust and quality; and usefulness	14
User version of the Mobile App Rating Scale	2 health apps	2016	English	—	5: engagement; functionality; esthetics; information; and subjective app quality	20
Health information technology usability evaluation scale	100 adults who tested the use of a mobile health app	2015	English	—	4: quality of work life; perceived usefulness; perceived ease of use; and user control	20
Measurement scales for perceived usefulness and perceived ease of use	2 studies: 112 users and 2 systems and 40 users and 2 systems	1998	English	—	2: perceived usefulness and perceived ease of use	12
The mHealth app usability questionnaire for interactive mHealth apps (patient version)	2 health apps	2019	English	—	3: Ease of use and satisfaction; System information arrangement; Usefulness	21
The mHealth app usability questionnaire for stand-alone mHealth apps (patient version)	2 health apps	2019	English	—	3: ease of use; interface and satisfaction; and usefulness	18
System Usability Scale	3 studies: 20 people, 206 studies using System Usability Scale, and 9000 System Usability Scale questionnaires	1986	English	Portuguese, Spanish, French, German, Persian, and Malay	1: usability	10

^a^Not analyzed.

#### Overall Quality

iSYScore, developed in 2015, was initially designed to measure the reliability and overall quality of mHealth apps, not only their usability [28]. It was validated according to 3 main aspects: popularity and interest, trust and quality, and usefulness [28]. Another important tool was the MARS developed in 2015 [29]. This scale has been adapted to Spanish and Italian languages [30]. It is worth noting that, in addition to its original version, this tool has a specific user version—user version of MARS (uMARS)—developed in 2016 [31]. Both the MARS versions (user and professional versions) focus on measuring other components of the quality of mHealth apps and not just usability, and they are widely used methods for measuring the quality of mHealth apps in different contexts [32-39]. The number of items and the different dimensions assessed by each mHealth measurement tool, as well as the main characteristics, are listed in Table 2.

### Quality Assessment: Psychometric Characteristics (COSMIN Analysis)

The main characteristics and the results of the quality analysis according to the psychometric properties of the measurement tools (COSMIN analysis) are summarized in Multimedia Appendix 1. Most of the studies did not assess or report all the properties recommended by COSMIN. Therefore, the information reflected in this review refers to the values of the properties reported in the original studies consulted.

## Discussion

### Principal Findings

The aim of this study is to review the literature and collect and analyze the tools used to assess the quality of mHealth apps. As described earlier, an objective criterion (analysis of psychometric characteristics through the COSMIN guideline) was used along with a subjective criterion (assessment of the adequacy of the number of dimensions and items evaluated by the tools). The main finding of this review was the generalized lack of validation of the psychometric characteristics of the available tools, including those most commonly used. In addition, there is no robust set of outcome measures for understanding the different dimensions of mHealth apps. For overall quality, the MARS scale seems to be a potentially valid tool to establish a standardized use of it.

### Validation of Psychometric Properties and Dimensions Included: 2 Characteristics to Consider When Choosing an Appropriate Tool

#### Usability Measurement

Regarding psychometric validation, 5 of the mHealth measurement tools included in this review met 4 or more of the properties recommended in the COSMIN guideline. The most validated tools are the *Measurement Scales for Perceived Usefulness and Perceived Ease of Use* and the Health-ITUES, with validation of 6 out of the 10 psychometric properties, and the SUS and the 2 versions of the MAUQ, with the validation of 4 properties each. However, these tools mainly focus on usability. Although usability is a critical aspect of an app that is expected to be used regularly, quality assessment cannot focus solely on this feature. This is worth highlighting because depending on whether the professional intends to assess only this specific dimension or requires further examination of mHealth apps, the choice of measurement tool based solely on the amount of validated psychometric properties may not be sufficient. The *Measurement Scales for Perceived Usefulness and Perceived Ease of Use* and the SUS are frequently used for evaluating mHealth apps; however, they are not mobile-specific. In contrast, the Health-ITUES and the MAUQ were explicitly designed for smartphones, as they allow one to evaluate the specific properties of this type of technology. The main limitation of Health-ITUES is that a unique mHealth app developed for community-dwelling adults living with HIV was used to validate the psychometric characteristics.

#### General Assessment

Considering the variety of dimensions evaluated, both MARS scales (professional and user versions) seem to be the tools that allow the most detailed measurement, assessing 5 aspects of the mHealth apps: engagement, functionality, esthetics, information quality, and subjective app quality. The evaluation of these additional characteristics of the apps allows for an in-depth analysis. Similarly, the MARS scale contains items related to the theoretical background, target population, or technical aspects of security or privacy of user data. However, these are not considered in the final score of the measurement instrument. Both user and professional versions have validated 3 essential psychometric properties with adequate results using around 60 mental health apps (content validity, evaluated by an expert panel to select the questionnaire items; internal consistency, Cronbach α=.90; and reliability, intraclass correlation=0.79 and 0.70 for professional and user versions, respectively). One of the strengths of this tool is the availability of a specific version for users (uMARS) with validation of the same psychometric characteristics (and with similar results) as the standard version. In addition, the MARS scale has been cross-culturally adapted and validated for different languages and is currently being adapted to other languages. There is abundant literature on the use of MARS for the evaluation of several mHealth apps, making it one of the most studied tools for assessing the quality of this kind of app.

The iSYScore tool is another method for measuring the overall quality of mHealth apps [28]. This tool has 2 significant disadvantages: it has insufficient validation (only content validity by an expert panel) and is only available in the Spanish language, so its use is severely limited.

### Availability of Different Versions of the Tools: Are They All Equally Studied?

#### Overview

Most of these tools focus on professional use. They seek to measure the usability or the overall quality of mHealth apps in an expert way [10,23,24,28]. However, allowing users of a specific population to evaluate the quality of apps designed for their use can allow one to analyze quality from another point of view, by dividing the beliefs and expectations of professionals and users. This requires the development of tools specifically designed for users or, at least, validated versions. Only 2 of the tools included in this review have a user version. The MARS [29] uses uMARS as the user version [31]. The MAUQ has 2 versions: the stand-alone mHealth app for patients and the interactive mHealth app for patients [25]. Therefore, one of the main strengths of the MAUQ tool is the availability of 4 versions of the questionnaire: interactive apps for patients, interactive apps for providers, stand-alone apps for patients, and stand-alone apps for providers. This fact allows greater versatility in the use of the questionnaire, which is specific for each type of user and app. However, only the 2 user versions have been tested and validated using 2 health apps, so the validity and reliability of the professional versions should still be studied.

#### Lack of Reliability of Self-created Questionnaires

The objective of this review was to use psychometrically validated measurement tools to assess mHealth apps. However, in the scientific literature, there are mainly 2 options used by authors in studies for this evaluation of mHealth apps. First, a large number of authors choose to use self-created questionnaires explicitly designed to evaluate the characteristics of their specific apps. Because it can be personalized, this option allows greater flexibility than other generic tools. However, most of these tools are used in studies that do not focus on their validation by recruiting small samples of participants that do not allow a reliable analysis of the psychometric characteristics. Consequently, although personalized questionnaires are frequently used to determine the usability and quality of app contents, their lack of validation and the lack of knowledge about their reliability raise questions about their suitability for use. Therefore, this type of tool was not included in this review. Second, many authors choose to use previously validated tools to maximize the reliability of the results obtained, despite the possible loss of personalization derived from the use of generic tools. Traditionally, there has been widespread use of tools designed for the technological environment in health, such as the SUS scale, which is still used today. However, the use of tools not specifically designed for mobile environments limits the analysis of specific characteristics of this type of technology. For this reason, it is essential to standardize the use of validated measurement tools designed explicitly for mobiles.

#### Developer Transparency and Data Privacy and Security

This review shows a lack of evaluation of relevant aspects of mHealth apps, such as developer transparency and policies regarding user data privacy and security [1]. Although some tools such as MARS incorporate items with some of these aspects, the fact that these are not included in the final scores of the available tools demonstrates the need to focus on this point.

### Strengths and Limitations

This is the first review to analyze the psychometric characteristics of the assessment tools of mHealth apps adhering to the COSMIN criteria. The main weakness is the difficulty in performing an optimal search and establishing adequate selection criteria because of the great heterogeneity in the tools and studies available in the literature. Another weakness is the possible risk of bias due to the possibility of losing a tool published in unreviewed databases. The implemented systematic methodology minimized biases derived from this situation.

Future studies should focus on creating and validating new tools or improving the validation of the most commonly used tools.

### Conclusions

In conclusion, although there is growing evidence about the use of tools to assess the quality and content of mHealth apps, the availability of specific, highly validated tools for mobile apps is still an unexplored topic in the market. There is no robust scorecard to understand the different dimensions of mHealth apps. The COSMIN guideline allows clinicians and scientific consensus to be brought closer to the field of digital health. According to the psychometric properties, the Health-ITUES scale and the *Measurement Scales for Perceived Usefulness and Perceived Ease of Use* were the most validated tools. However, the validation is specific to a single app from a field, and its design is focused on evaluating its usability aspects. The MARS tool obtained adequate outcomes in a moderate number of psychometric characteristics, and it has been validated in a large number of mHealth apps. Its current use is widespread and evaluates different aspects of the app quality, as well as its usability.

This review suggests that the continuation of the validation of this tool in other psychometric properties might provide an appropriate option for evaluating the quality of mHealth apps that is requested by the market in the long term to quickly identify relevant apps.

## Appendix

Multimedia Appendix 1 Results of the quality analysis of the measurement tools according to their psychometric properties (Consensus-based Standards for the Selection of Health Measurement Instruments).

## Abbreviations

COSMIN: Consensus-based Standards for the Selection of Health Measurement Instruments
Health-ITUES: Health Information Technology Usability Evaluation Scale
MARS: Mobile App Rating Scale
MAUQ: mHealth Apps Usability Questionnaire
mHealth: mobile health
PRISMA: Preferred Reporting Items for Systematic Reviews and Meta-Analyses
SUS: System Usability Scale
uMARS: user version of the Mobile App Rating Scale
