# Supplementary material for: Tools for Evaluating the Content, Efficacy, and Usability of Mobile Health Apps According to the Consensus-Based Standards for the Selection of Health Measurement Instruments: Systematic Review
Source: JMIR Mhealth Uhealth. 2021 Dec 1;9(12):e15433. doi: 10.2196/15433 (PMC8686474; doi:10.2196/15433)
Supplement: Multimedia Appendix 1 [file mhealth_v9i12e15433_app1.docx]

**MULTIMEDIA APPENDIX**

**Multimedia appendix 1.** Results of the quality analysis of the measurement tools according to their psychometric properties (Consensus-based Standards for the Selection of Health Measurement Instruments).

| Measurement tool | Psychometric properties | | | | | | | | | | |
| --- | --- | --- | --- | --- | --- | --- | --- | --- | --- | --- | --- |
|  | Internal consistency, Cronbach α | Reliability, ICC^a^ (95% CI) | Measurement error | Content validity | Structural validity | Hypotheses testing | Cross-cultural validity | Criterion validity | Responsiveness | Interpretability | Number of properties satisfied (N=10), n (%) |
|  | | | | | | | | | | | |
| Mobile App Rating Scale | .90 | 0.79 (0.75-0.83) | NO^b^ | Expert panel to select the questionnaire items | NO | NO | NO | NO | NO | NO | 3 (30) |
| iSYScore index | NO | NO | NO | Expert panel to select the questionnaire items | NO | NO | NO | NO | NO | NO | 1 (10) |
| User version of the Mobile App Rating Scale | .90 | 0.66^c^ and 0.70^d^ | NO | Expert panel and focus groups (164 young people) to select the questionnaire items | NO | NO | NO | NO | NO | NO | 3 (30) |
| Health information technology usability evaluation Scale | .85-.92 | NO | NO | Expert panel and factor analysis to select the questionnaire items | EFA^e^ and CFA^f^ | The SEM^g^ hypothesis was accepted. In other words, the general factor and *usability* can predict *intention to use* | NO | Correlation with the Poststudy System usability questionnaire | Statistically significant difference was demonstrated with intervention group | NO | 6 (60) |
| Measurement scales for perceived usefulness and perceived ease of use | .97^h^ and .91^i^ | NO | .08 | Focus groups to select the questionnaire items | EFA | Correlations between perceived usefulness and ease of use with self-reported indicants of system use | NO | Convergent and discriminant validity were tested | NO | NO | 6 (60) |
| The mHealth app usability questionnaire for interactive mHealth apps (patient version)^j^ | .895, .829, and .900 | NO | NO | Expert panel (7 experts) to select the questionnaire items | EFA | NO | NO | Correlation with the Poststudy System Usability Questionnaire and the System Usability Scale | NO | NO | 4 (40) |
| The mHealth app usability questionnaire for stand-alone mHealth apps (patient version)^k^ | .847, .908, and .717 | NO | NO | Expert panel (7 experts) to select the questionnaire items | EFA | NO | NO | Correlation with the Poststudy System Usability Questionnaire and the System Usability Scale | NO | NO | 4 (40) |
| System Usability Scale | .911 | NO | NO | Focus group of 20 people to select the questionnaire items | CFA and EFA | Correlation of *r*=0.806 between the System Usability Scale and a single 7-point adjective rating question for an overall rating of *user friendliness* | NO | NO | NO | NO | 4 (40) |

^a^ICC: intraclass correlation.

^b^Not analyzed.

^c^Over a 1-2-month period.

^d^Over a 3-month period.

^e^EFA: exploratory factor analysis.

^f^CFA: confirmatory factor analysis.

^g^SEM: SE of the mean.

^h^For usefulness.

^i^For ease of use.

^j^For the 3 subscales.

^k^For the 3 subscales.
